# Post-marketing safety surveillance of dalfampridine for multiple sclerosis using FDA adverse event reporting system

**DOI:** 10.3389/fphar.2023.1226086

**Published:** 2023-09-14

**Authors:** Rui Xiong, Jing Lei, Sicen Pan, Hong Zhang, Yongtao Tong, Wei Wu, Yi Huang, Xiaodan Lai

**Affiliations:** ^1^ Department of Pharmacy, The 958th Hospital of Chinese People’s Liberation Army, Chongqing, China; ^2^ Department of Pharmacy, Daping Hospital, Army Medical University, Chongqing, China; ^3^ Department of Pharmacy, The 956th Hospital of Chinese People’s Liberation Army, Nyingchi, Tibet, China; ^4^ Biomedical Analysis Center, Army Medical University, Chongqing, China

**Keywords:** FAERS, dalfampridine, multiple sclerosis, adverse event, pharmacovigilance

## Abstract

**Objective:** To investigate and analyze the post-marketing adverse event (AE) data of multiple sclerosis (MS) therapeutic drug dalfampridine using the US Food and Drug Administration Adverse Event Reporting System (FAERS) for its clinical safety application.

**Methods:** Use OpenVigil2.1 platform to obtain AE data of dalfampridine from FAERS from February 2010 to September 2022. Match “adverse drug reaction” with preferred term (PT) and system organ class (SOC) according to the Medical Dictionary for Regulatory Activities (MedDRA), then merge the same PT and delete non-AE PT. Positive signals were identified by the reporting odds ratio (ROR), proportional reporting ratio (PRR), and Bayesian confidence propagation neural network (BCPNN) methods. When AE signals met the criteria of those three methods, they were identified as positive signals.

**Results:** A total of 44,092 dalfampridine-related AE reports were obtained, and 335 AE signals were identified, including 11,889 AE reports. AEs were more common in females and in the 45–65 age group, which is consistent with the epidemiological characteristics of MS. The 335 AE signals involved 21 SOCs, including investigations, infections and infestations, eye disorders, etc. Among the top 20 PTs in signal strength, 10 were associated with abnormal lymphocyte percentage and count, and 5 were associated with abnormal urine tests, some of which were not described in the instruction, such as spinal cord injury cauda equina, haemoglobin urine present, urinary sediment abnormal and so on. The most frequently reported AE signals were urinary tract infection, dizziness, condition aggravated. In addition, 23 AE signals with death outcomes were identified, with an incidence of less than 0.1%.

**Conclusion:** Data mining of FAERS was conducted to analyze the AEs of dalfampridine, and new AE signals were found. This study provides a reference for the safe use of dalfampridine in the treatment of MS.

## 1 Introduction

Multiple sclerosis (MS) is an autoimmune central nervous system (CNS) disorder characterized by inflammatory demyelination and axonal transection, defined as severed terminal axonal structures representing the pathological correlate of irreversible neurologic damage (McGinley et al., 2021). MS is a process that progresses from an at-risk state through asymptomatic, prodromic, and symptomatic stages, and axonal and neuronal loss begins at the early stages of the disease process, resulting in cognitive impairment and other early disability (Katz Sand, 2015). Typical clinical manifestations of MS include unilateral optic neuritis (blurred vision with associated pain), partial myelitis (extremity and torso impaired sensation, weakness, and/or ataxia), focal sensory disturbance (limb paresthesias, abdominal or chest banding dysesthesia), or brainstem syndromes (intranuclear ophthalmoplegia, vertigo, hearing loss, facial sensory disturbance) (Dobson and Giovannoni, 2019; McGinley et al., 2021). The worldwide prevalence of MS ranges from 5 to 300 per 100,000 people, rising with increasing latitude, and is more common in young and middle-aged adults (mean age of diagnosis is 20–30 years) (Browne et al., 2014; Howard et al., 2016). The underlying cause of MS remains uncertain, but many genetic (e.g., major histocompatibility complex HLA-DRB1 locus) and environmental factors such as vitamin D levels, environmental UV radiation, Epstein-Barr virus infection, and smoking have been reported to be associated with MS (Dobson and Giovannoni, 2019).

Currently, there is no curative treatment available for MS, and its treatment mainly includes acute relapses therapies, which are mostly corticosteroids, disease-modifying therapies (DMTs), which mainly includes β-interferon, glatiramer acetate, teriflunomide, fingolimod, ozanimod, natalizumab, ofatumumab, etc., and symptomatic therapies, which aimed to manage the complications associated with MS (Zhang et al., 2021; Travers et al., 2022). Although DMTs are effective in reducing the risk of relapse and potentially disability, they cannot address the poor quality of life or stop disease progression.

Dalfampridine (4-aminopyridine) is a voltage-dependent potassium channel blocker that acts on potassium channels exposed in MS patients to restore conduction in local demyelinating axons (Ghebleh Zadeh et al., 2019). Dalfampridine also promotes calcium (Ca^2+^) influx at presynaptic terminals, thereby enhancing the neuronal or neuromuscular transmission of normal myelin neurons (Zhang et al., 2021). These pharmacological properties suggest its therapeutic potential in neuromuscular transmission disorders and demyelinating diseases. In January 2010, dalfampridine was approved by the United States Food and Drug Administration (FDA) to improve walking speed and distance in MS patients. Dalfampridine, also known as fampridine in Europe, was conditionally approved for marketing by the European Medicines Agency (EMA) in July 2011 and fully approved for marketing in 2017, and is now available in Germany, the United Kingdom, France and other countries (Guo et al., 2016). Although dalfampridine has been approved for marketing in dozens of countries, treatment has been mainly limited to patients in western countries, and the drug is rarely included as a potential therapeutic option for the symptomatic treatment of MS in other countries or regions such as Latin America and Asia, which may be related to the limited financial resources allocated to healthcare in developing countries and the relatively high cost of treatment in dalfampridine (Zhang et al., 2021). Despite many clinical studies have demonstrated its positive efficacy, adverse reactions of dalfampridine, such as paresthesias, dizziness, anxiety, insomnia, and confusion, have troubled some patients (Goodman et al., 2009; Goodman et al., 2010; Hobart et al., 2019). In order to better apply dalfampridine, it is necessary to further explore and analyze the adverse events (AEs) caused by dalfampridine to reduce or avoid the occurrence of AEs.

Although many adverse reactions have been described in the instruction and in some clinical studies, the adverse reactions of dalfampridine may not be fully revealed due to sample size and ethical limitations. Real-world data contribute to a more comprehensive understanding of the safety of dalfampridine, and the FDA Adverse Event Reporting System (FAERS) is a representative AE database that provides a good paradigm for pharmacovigilance studies. In this study, AEs of dalfampridine were mined through the FAERS to provide an overall understanding of the safety of dalfampridine.

## 2 Materials and methods

### 2.1 Data sources

Data in this study were obtained from the FAERS through OpenVigil2.1, a software package for analyzing pharmacovigilance data (Bohm et al., 2021). Using drug name “Dalfampridine” as the keyword, the search time range was 1 February 2010 to 30 September 2022, and the minimum age of patients was limited to 18 years. By setting filter conditions such as gender, age, country, year and outcome, AE reports of dalfampridine were extracted respectively for subgroup analysis. The AE report outcomes included death, congenital anomaly, disability, life-threatening, hospitalization (initial or prolonged), required intervention to prevent permanent impairment, and others, with the first six being severe AE outcomes.

### 2.2 Data processing

The classification and standardization of AEs in FAERS data is referred to the Medical Dictionary for Regulatory Activities (MedDRA) (Tian et al., 2022). In the FAERS database, each report is coded using Preferred Terms (PTs) from MedDRA. In MedDRA, a given PT can be assigned to a specific High-level Term (HLT), High-level Group Term (HLGT), and System Organ Class (SOC) level, but each HLT, HLGT, and SOC often contains multiple PT. This study analyzed the data derived from OpenVigil2.1 platform under the definition of MedDRA. Standardize the PT of all AE terms through the MedDRA (version 26.0) and then merge the same PT entries. In addition, non-drug AE terms and AE terms associated with MS symptoms and indications for dalfampridine were removed. Then, a two-by-two contingency table was constructed (Table 1), and disproportional AEs and drug combinations were identified.

**TABLE 1:** Two-by-two contingency table for disproportionality analyses.

Event groups	Drug used	Other drugs	Sums
Event	*a*	*c*	*a+c*
Other events	*b*	*d*	*b + d*
Sums	*a+b*	*c + d*	*a+b + c + d*

### 2.3 AE signal detection

Reporting odds ratio (ROR), United Kingdom medicines and healthcare products regulatory agency (MHRA), and Bayesian confidence propagation neural network (BCPNN) methods were used to detect AE signals. ROR, proportional reporting ratio (PRR) and information component (IC) values were calculated according to values a, b, c, and d in Table 1, and positive signals were identified according to the criteria in Table 2. Only AEs that meet the thresholds of all three methods are identified as positive AE signals. Microsoft Excel 2019 and Graphpad prism 8.0 software were used for data analysis.

**TABLE 2:** Calculation formulas and thresholds of ROR, PRR, and BCPNN methods.

Methods	Calculation formula	Threshold
ROR	ROR = (*a*/*c*)/(*b*/*d*)	*a* ≥ 3 and 95% CI lower limit of ROR >1
	95% CI = $e^{lnROR\pm 1.96\sqrt{\frac{1}{a}+\frac{1}{b}+\frac{1}{c}+\frac{1}{d}}}$	
MHRA	PRR = [*a*(*a*+*b*)]/[*c*(*c* + *d*)]	*a* ≥ 3, PRR ≥2 and χ^2^ ≥ 4
	χ^2^ = $\frac{\left(a+b+c+d\right){\left(ad-bc\right)}^{2}}{\left(a+c\right)\left(a+b\right)\left(c+d\right)\left(b+d\right)}$	
BCPNN	IC = $\log_{2}\frac{a\left(a+b+c+d\right)}{\left(a+b\right)\left(a+c\right)}$	*a* ≥ 3 and IC-2SD > 0
	IC-2SD = $\log_{2}\frac{\left(a+\gamma_{11}\right)\left(N+\alpha \right)\left(N+\beta \right)}{\left(N+\gamma \right)\left(a+b+\alpha_{1}\right)\left(a+c+\beta_{1}\right)}-2\sqrt{V\left(IC\right)}$	
	*V(IC)* = ${\left(\frac{1}{\log\;2}\right)}^{2}$ [ $\frac{N-a+\gamma -\gamma_{11}}{\left(a+\gamma_{11}\right)\left(1+N+\gamma \right)}+\frac{N-a-b+\alpha -\alpha_{1}}{\left(a+b+\alpha_{1}\right)\left(1+N+\alpha \right)}+\frac{N-a-c+\beta -\beta_{1}}{\left(a+c+\beta_{1}\right)\left(1+N+\beta \right)}$ ]	
	$\gamma =\gamma_{11}\frac{\left(N+\alpha \right)\left(N+\beta \right)}{\left(a+b+\alpha_{1}\right)\left(a+b+\beta_{1}\right)}$	
	$\gamma_{11}=1$ , $\alpha_{1}=\beta_{1}=1$ , α=β=2 , N=a+b+c+d	

## 3 Results

### 3.1 Descriptive analysis

**FIGURE 1:**
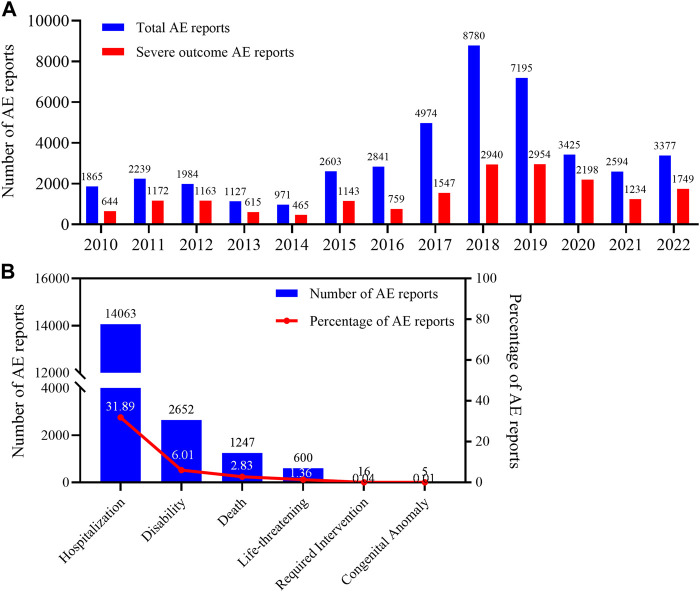
The annual distribution of AE reports and the composition of severe outcome AE reports for dalfampridine. **(A)** Annual distribution of AE reports, **(B)** The types, cases and proportion of severe outcome AE. Since the reporting years of some AE reports are not clear, the total AE in the Figure 1A shows only 43975.

From February 2010 to September 2022, a total of 4,541,880 AE reports were retrieved from the FAERS using the OpenVigil2.1 platform, of which 44,092 were AE reports with dalfampridine as the primary suspect drug, accounting for about 0.97%. By subgroup analysis of gender, age and country, we found that the patient gender in these AE reports was mainly female, accounting for 74.68%, the age of the patients was mainly 46–65, accounting for 63.91%, and the reporting country was mainly United States, accounting for 84.30% (Table 3). The annual distribution of AE reports showed a trend of increasing first and then decreasing, and the distribution of years reported by AEs with severe outcomes showed similar characteristics. (Figure 1A). In 2018, the number of AE reports reached 8780, among which 2940 were severe outcomes. There were 18,583 AE reports with severe outcomes, accounting for 42.15% of all reports, and hospitalization is the main severe outcome, accounting for 31.89%, while required intervention and congenital anomaly are sporadic, accounting for less than 0.1% (Figure 1B).

**TABLE 3:** Basic information about AE reports of dalfampridine from February 2010 to September 2022.

Entry	AE number	Percentage (%)
Gender of patient		
Male	11019	24.99
Female	32929	74.68
Unknown	144	0.33
Age		
18–45	8899	20.18
46–65	28177	63.91
>65	6968	15.80
Unknown	48	0.11
Reporter country		
United Sates	37170	84.30
Germany	1924	4.36
Canada	1646	3.73
France	426	0.97
United Kingdom	124	0.28
Australia	93	0.21
Others and unknown	2709	6.14

### 3.2 Disproportionality analysis of AEs at the SOC level

By excluding and combining the PT entries, a total of 335 AE signals were identified, including 11,889 AE reports. These 335 AE signals were classified according to the corresponding SOC of MedDRA involving 21 SOCs. We evaluated the correlation between AEs and organs by ROR, MHRA, and BCPNN methods. The larger the ROR, PRR, and IC values, the stronger the correlation. The results showed that the SOCs with the strongest relevance were investigations, infections and infestations, eye disorders, and so on (Table 4). The SOCs containing the most positive signals are investigations (117), nervous system disorders (47), injury, poisoning and procedural complications (40), infections and infestations (27), and renal and urinary disorders (25) respectively, and eye disorders, skin and subcutaneous tissue disorders, cardiac disorders, and immune system disorders all contain only one positive signal. Among them, 6 SOCs, such as metabolism and nutrition disorders, reproductive system and breast disorders, injury, poisoning and procedural complications, neoplasms benign, malignant and unspecified (incl cysts and polyps), endocrine disorders, and ear and labyrinth disorders, were not reported in the instruction.

**TABLE 4:** The SOC of AEs associated with dalfampridine (Sort by IC value).

SOC name	PT number	N (%)	ROR (95% CI)	PRR (χ^2^)	IC (IC-2SD)
Investigations	117	2181 (18.34)	13.49 (12.88, 14.14)	11.47 (20,447.67)	3.48 (3.26)
Infections and infestations	27	1511 (12.71)	9.61 (9.11, 10.15)	8.65 (10,096.25)	3.08 (2.85)
Eye disorders	1	3 (0.03)	7.84 (2.49, 24.62)	7.83 (17.48)	2.94 (2.29)
Metabolism and nutrition disorders	4	47 (0.40)	5.93 (4.44, 7.92)	5.92 (188.82)	2.54 (2.18)
Blood and lymphatic system disorders	3	61 (0.51)	5.57 (4.32, 7.17)	5.54 (223.73)	2.45 (2.10)
Vascular disorders	4	380 (3.20)	4.70 (4.24, 5.21)	4.60 (1062.07)	2.19 (1.92)
Renal and urinary disorders	25	795 (6.69)	4.77 (4.44, 5.13)	4.55 (2198.69)	2.17 (1.93)
Gastrointestinal disorders	4	33 (0.28)	4.15 (2.94, 5.85)	4.14 (77.64)	2.04 (1.65)
Skin and subcutaneous tissue disorders	1	18 (0.15)	3.75 (2.35, 5.96)	3.74 (35.80)	1.89 (1.46)
Reproductive system and breast disorders	2	10 (0.08)	3.70 (1.99, 6.91)	3.70 (19.51)	1.88 (1.40)
Injury, poisoning and procedural complications	40	1103 (9.28)	3.87 (3.64, 4.11)	3.63 (2129.85)	1.85 (1.61)
Neoplasms benign, malignant and unspecified (incl cysts and polyps)	8	58 (0.49)	3.56 (2.74, 4.61)	3.54 (104.98)	1.81 (1.46)
Respiratory, thoracic and mediastinal disorders	2	20 (0.17)	3.43 (2.21, 5.33)	3.42 (33.98)	1.77 (1.34)
Nervous system disorders	47	3411 (28.69)	4.13 (3.97, 4.29)	3.34 (5983.38)	1.73 (1.52)
General disorders and administration site conditions	14	976 (8.21)	3.42 (3.21, 3.66)	3.25 (1539.10)	1.69 (1.45)
Endocrine disorders	2	11 (0.09)	3.14 (1.73, 5.69)	3.14 (15.90)	1.64 (1.17)
Psychiatric disorders	14	694 (5.84)	2.95 (2.74, 3.19)	2.85 (844.00)	1.50 (1.26)
Cardiac disorders	1	11 (0.09)	2.85 (1.57, 5.16)	2.85 (13.09)	1.50 (1.03)
Musculoskeletal and connective tissue disorders	16	464 (3.90)	2.46 (2.24, 2.70)	2.41 (385.23)	1.26 (1.00)
Ear and labyrinth disorders	2	93 (0.78)	2.17 (1.77, 2.67)	2.17 (58.12)	1.11 (0.78)
Immune system disorders	1	9 (0.08)	2.15 (1.12, 4.14)	2.15 (5.48)	1.10 (0.61)

% indicates the proportion of corresponding AE, reports in the total reports of positive AE, signals (11,889 cases).

### 3.3 Disproportionality analysis of AEs at the PT level

The top 20 PTs of AE with the strongest relevance with dalfampridine are shown in Table 5, including spinal cord injury cauda equina, CD8 lymphocyte percentage decreased, haemoglobin urine present, etc. Among them, spinal cord injury cauda equina, haemoglobin urine present, urine leukocyte esterase positive, urinary sediment abnormal and specific gravity urine abnormal are AEs not mentioned in the instruction. In addition, as is shown in Supplementary Table S1, the highest number of AE reported included urinary tract infection (1061, 8.92%), dizziness (764, 6.43%), condition aggravated (606, 5.10%), etc., which were also the main adverse reactions reported in the instruction. In order to further understand the age, gender and countries/regions distribution of common AE, we conducted subgroup analysis of AE in the top 3 reported AE numbers. As shown in Table 6, the rates of urinary tract infection, dizziness, condition aggravated are all above 72% in females and above 60% in the 46–65 age group, and the rates reported by the United States are above 86%.

**TABLE 5:** Top 20 PTs of signal strength of AEs associated with dalfampridine (Sort by IC value).

PT name	N (%)	ROR (95% CI)	PRR (χ^2^)	IC (IC-2SD)
Spinal cord injury cauda equina	6 (0.05)	1011.04 (204.04, 5009.82)	1010.59 (1512.88)	7.99 (6.99)
CD8 lymphocyte percentage decreased	3 (0.03)	1010.81 (105.13, 9718.49)	1010.59 (756.44)	7.99 (6.31)
Haemoglobin urine present	17 (0.14)	337.29 (172.16, 660.78)	336.86 (2846.37)	7.40 (6.84)
CD8 lymphocytes increased	6 (0.05)	337.01 (108.68, 1045.08)	336.86 (1004.60)	7.40 (6.56)
Somatosensory evoked potentials abnormal	6 (0.05)	288.87 (97.07, 859.66)	288.74 (926.40)	7.28 (6.46)
CD4 lymphocyte percentage decreased	6 (0.05)	252.76 (87.69, 728.58)	252.65 (859.38)	7.18 (6.38)
Urine leukocyte esterase positive	51 (0.43)	233.04 (163.06, 333.06)	232.16 (6949.36)	7.11 (6.70)
B-lymphocyte count abnormal	11 (0.09)	231.78 (107.55, 499.53)	231.59 (1496.64)	7.10 (6.49)
B-lymphocyte count decreased	61 (0.51)	192.91 (140.80, 264.32)	192.04 (7383.87)	6.94 (6.55)
T-lymphocyte count increased	17 (0.14)	191.13 (105.39, 346.61)	190.89 (2049.72)	6.93 (6.41)
CD4 lymphocytes increased	15 (0.13)	174.43 (93.50, 325.42)	174.24 (1702.89)	6.85 (6.31)
Vitamin B12 abnormal	8 (0.07)	158.62 (68.44, 367.60)	158.52 (851.53)	6.76 (6.10)
CD8 lymphocytes decreased	14 (0.12)	152.29 (81.00, 286.33)	152.13 (1448.01)	6.72 (6.18)
Lymphocyte percentage abnormal	3 (0.03)	144.40 (37.34, 558.49)	144.37 (298.99)	6.66 (5.62)
Urinary sediment abnormal	5 (0.04)	129.61 (46.20, 363.61)	129.56 (460.67)	6.55 (5.79)
Urine analysis abnormal	179 (1.51)	124.45 (104.79, 147.80)	122.81 (15,850.60)	6.50 (6.19)
Cystitis *klebsiella*	8 (0.07)	112.35 (50.47, 250.13)	112.29 (661.78)	6.40 (5.78)
Specific gravity urine abnormal	3 (0.03)	112.31 (30.40, 414.91)	112.29 (248.17)	6.40 (5.42)
Culture urine positive	58 (0.49)	94.34 (70.48, 126.27)	93.93 (4170.19)	6.20 (5.83)
T-lymphocyte count decreased	27 (0.23)	92.99 (60.71, 142.43)	92.81 (1922.52)	6.19 (5.75)

% indicates the proportion of corresponding AE, reports in the total reports of positive AE, signals (11,889 cases).

**TABLE 6:** Age, gender and country/regional distribution of the top 3 PT.

Entry name	Urinary tract infection N (%)	Dizziness N (%)	Condition aggravated N (%)
Gender of patient			
Male	175 (16.49)	156 (20.42)	164 (27.06)
Female	882 (83.13)	605 (79.19)	440 (72.61)
Unknown	4 (0.38)	3 (0.39)	2 (0.33)
Age			
18–45	157 (14.80)	153 (20.03)	110 (18.15)
46–65	694 (65.41)	459 (60.08)	378 (62.38)
>65	207 (19.51)	150 (19.63)	118 (19.47)
Unknown	3 (0.28)	2 (0.26)	-
Reporter country/region			
United Sates	983 (92.65)	658 (86.13)	566 (93.40)
European	36 (3.39)	—	—
Canada	12 (1.13)	—	—
Others	30 (2.83)	106 (13.87)	40 (6.60)

% indicates the proportion of the entry to the corresponding total cases.

### 3.4 AE analysis of death outcome

In order to understand the severe AE with a death outcome, 23 AE signals were identified according to the criteria in Table 2, and the results were shown in Table 7. Among them, pneumonia aspiration (12, 1.01‰), urinary tract infection (11, 0.93‰), progressive multifocal leukoencephalopathy (9, 0.76‰) and seizure (9, 0.76‰) have the largest number of reports. Erosive duodenitis, haemorrhagic erosive gastritis, duodenitis, osteoporosis, intracranial pressure increased, decubitus ulcer, lung cancer metastatic, transient ischaemic attack, aphasia, dysarthria, lung carcinoma cell type unspecified stage IV, and pneumonia aspiration are not mentioned in the instruction.

**TABLE 7:** The PT of AEs with a death outcome (Sort by IC value).

PT name	N (‰)	ROR (95% CI)	PRR (χ^2^)	IC (IC-2SD)
Erosive duodenitis	3 (0.25)	253.72 (71.37, 901.98)	252.13 (600.34)	7.66 (5.14)
Haemorrhagic erosive gastritis	3 (0.25)	234.20 (66.52, 824.52)	232.74 (562.45)	7.56 (5.07)
Duodenitis	3 (0.25)	59.69 (18.57, 191.93)	59.33 (162.49)	5.81 (3.50)
Central nervous system lesion	5 (0.42)	30.50 (12.48, 74.59)	30.20 (137.09)	4.88 (2.70)
Immune reconstitution inflammatory syndrome	3 (0.25)	17.10 (5.44, 53.71)	17.00 (44.44)	4.06 (1.81)
Paraesthesia	5 (0.42)	14.14 (5.82, 34.35)	14.01 (59.61)	3.79 (1.63)
Progressive multifocal leukoencephalopathy	9 (0.76)	14.19 (7.31, 27.57)	13.94 (106.80)	3.78 (1.68)
Osteoporosis	3 (0.25)	13.90 (4.43, 43.58)	13.82 (35.20)	3.77 (1.52)
Intracranial pressure increased	3 (0.25)	12.27 (3.92, 38.44)	12.20 (30.49)	3.59 (1.35)
Decubitus ulcer	4 (0.34)	11.72 (4.35, 31.52)	11.63 (38.43)	3.52 (1.33)
Ill-defined disorder	4 (0.34)	10.06 (3.74, 27.05)	9.99 (32.05)	3.31 (1.12)
Hypoaesthesia	5 (0.42)	9.71 (4.01, 23.55)	9.62 (38.31)	3.25 (1.09)
Lung cancer metastatic	4 (0.34)	9.45 (3.52, 25.40)	9.38 (29.70)	3.22 (1.03)
Transient ischaemic attack	3 (0.25)	9.33 (2.98, 29.19)	9.28 (21.98)	3.20 (0.96)
Aphasia	4 (0.34)	8.00 (2.98, 21.49)	7.94 (24.10)	2.98 (0.79)
Urosepsis	3 (0.25)	5.94 (1.90, 18.55)	5.91 (12.18)	2.56 (0.32)
Dysarthria	3 (0.25)	5.77 (1.85, 18.02)	5.74 (11.69)	2.51 (0.28)
Lung carcinoma cell type unspecified stage IV	3 (0.25)	5.56 (1.78, 17.35)	5.53 (11.09)	2.46 (0.23)
Seizure	9 (0.76)	5.29 (2.73, 10.25)	5.21 (30.58)	2.38 (0.28)
Pneumonia aspiration	12 (1.01)	5.05 (2.84, 8.96)	4.95 (37.78)	2.30 (0.23)
Gastrointestinal disorder	3 (0.25)	4.95 (1.59, 15.45)	4.93 (9.36)	2.30 (0.06)
Urinary tract infection	11 (0.93)	4.88 (2.68, 8.89)	4.79 (33.03)	2.26 (0.18)
Metastases to central nervous system	4 (0.34)	4.61 (1.72, 12.37)	4.58 (11.18)	2.19 (0.01)

‰ indicates the proportion of corresponding AE, in the total AE, cases (11,889 cases).

## 4 Discussion

Mobility impairment is one of the most widespread and serious consequences of MS, which has many adverse effects on emotional wellbeing, activities of daily living, quality of life of patients (Heesen et al., 2008; Larocca, 2011). Studies have shown that 45% of patients report mobility problems within the first month of diagnosis, and more than 90% report mobility problems within 10 years of diagnosis (Baird et al., 2018). Therefore, improving walking ability has positive significance for improving the quality of life of MS patients. Dalfampridine is the first symptomatic pharmacologic agent approved by the FDA to improve walking in patients with MS (Baird et al., 2018). In this study, AE signals of dalfampridine were mined on the basis of real-world data, aiming to provide a comprehensive understanding of the safety of dalfampridine. Since dalfampridine was first approved in January 2010, we collected AE reports submitted to FAERS from February 2010 to September 2022, and a total of 44,092 AE reports were obtained, 74.68% of which were women, which was close to the gender ratio of MS patients, as women are two to three times more frequently affected than men (Oh et al., 2018). However, different from the peak incidence of 20–40 years old, the age group with the most AE reported was 45–65 years old, accounting for more than 65%, suggesting that older patients or patients with longer course of disease were more likely to suffer drug-induced damage. Fewer AEs were reported in patients over 65 years of age, possibly due to the higher mortality rate and shorter life expectancy of MS (Thormann et al., 2017). Among reporting countries or regions, the United States reported the largest number of AE, accounting for 84.30%, followed by Germany (4.36%) and Canada (3.73%), which is related to the high prevalence among white person, especially those of northern European descent (Ascherio and Munger, 2016). Another important reason is that the FAERS was established by the FDA in the United States and may be less used in other countries or regions. In addition, it may be related to the number of approved countries and the popularity of dalfampridine. From the perspective of the number of AE changes with the years, the number of AE reports showed a slight fluctuation from 2010 to 2016, reached a peak in 2017–2019, and then gradually fall back. It may be because fewer patients applied dalfampridine at the initial stage of its marketing. With the increase of clinical application of dalfampridine, the number of AE reports increased, and the decrease in the number of AE reports in recent years may be due to the fact that medical staff have a relatively full understanding of the safety of dalfampridine and have avoided some AE. The annual distribution of severe outcome AE reports was consistent with the change trend of total AE reports, accounting for about 42.15%. We also noted that the proportion of AE reports with serious outcomes fluctuated from year to year, which may be related to the continuous approval of dalfampridine in different countries, because the current safety studies on dalfampridine are mainly focused on Western countries and Caucasian populations, and there is less experience in other countries and ethnicities (Zhang et al., 2021). In addition, the outcome of AE report can only reflect the outcome of the patient with the corresponding AE, but whether these outcomes are caused by dalfampridine-induced adverse reaction or by disease progression remains to be distinguished.

In the current study, the AE signal of dalfampridine involved 21 SOC items, among which the strongest correlation was in investigations, suggesting that dalfampridine had a potential impact on investigations. The largest number of AE reports were for nervous system disorders, including dizziness, paresthesia, epilepsy, etc., which was consistent with the instruction of dalfampridine and may be related to its pharmacological mechanism of altering neuronal conduction or neuromuscular transmission (Pikoulas and Fuller, 2012). However, there are some nervous system disorders such as cognitive disorder, neuralgia and spinal cord disorder (Supplementary Table S2) that may also be associated with the progression of MS (Katz Sand, 2015; Dobson and Giovannoni, 2019). Therefore, clinicians should accurately identify the neurological symptoms of patients during dalfampridine treatment and take necessary measures. In our analysis, there are 6 SOCs that are not mentioned in the instruction, namely, metabolism and nutrition disorders, reproductive system and breast disorders, injury, poisoning and procedural complications, neoplasms benign, malignant and unspecified (incl cysts and polyps), endocrine disorders, and ear and labyrinth disorders. It is suggested that dalfampridine may have potential adverse effects on these systems and should be paid attention to in clinical use.

In the PT item analysis, the most significant AE signal was spinal cord injury cauda equina, which may be related to disease progression, as spinal cord injury is common in MS patients. However, an early study showed that electrical conduction of the spinal cord from the sixth cervical spine (C6) to the first lumbar spine (L1) was slowed, while the cauda equina was unaffected (Snooks and Swash, 1985). Therefore, whether the spinal cord injury cauda equina is caused by dalfampridine or MS progression needs further study. For many years, MS has been considered to be an autoimmune disease of the central nervous system mediated by T lymphocytes, triggered by environmental factors on the basis of genetic susceptibility genes (Oh et al., 2018). CD4^+^ T cells may play an important role in peripheral immune interactions leading to MS, while CD8^+^ T cells are the predominant T-cell population in brain lesions in patients with MS, and the number of CD8^+^ T cells is most correlated with the degree of axonal damage (Bar-Or and Li, 2021). Of the top 20 significant AE signals, 10 entries were associated with lymphocyte count or percentage abnormalities, including increased, decreased, or abnormal. Although abnormal lymphocyte counts or percentages are often associated with MS pathology, they are usually elevated rather than decreased, so excessive lymphocyte depletion may be an adverse outcome of dalfampridine. There were six signals associated with abnormal urine tests, which are likely related to kidney injury or urinary tract infection, consistent with the instructions and previous reports (Pikoulas and Fuller, 2012; Frejo et al., 2014; Zhong et al., 2017). Studies have shown that the plasma concentration (*C*_max_) of dalfampridine and area under the plasma concentration-time curve (AUC) in mild and severe renal impairment are 166.5%–199.9% and 175.3%–398.7% of healthy individuals, respectively, and the mean terminal disposition half-life was 6.4 h in healthy individuals, compared with 7.4, 8.1, and 14.3 h in patients with mild, moderate, and severe renal impairment, respectively (Smith et al., 2010). This suggests that dalfampridine should be used with caution in patients with mild and moderate renal impairment and should be contraindicated in patients with severe renal impairment. Previous studies have shown decreased levels of vitamin B12 in MS patients, and appropriately elevated levels of vitamin B12 may be beneficial for anti-inflammatory and myelin regeneration (Miller et al., 2005; Nemazannikova et al., 2018). This suggests that vitamin B12 abnormal is not AE signals of dalfampridine. In terms of the number of reported AE, urinary tract infection, dizziness and condition aggravated were the most frequently reported AE. Subgroup analysis showed that the percentage of female patients reporting urinary tract infection and dizziness was higher than the proportion of female in the total number of AEs, suggesting that women were more prone to urinary tract infection and dizziness. The percentages of patients aged 18–45 years old reporting urinary tract infection and the percentages of patients aged 45–65 years old reporting dizziness and condition aggravated were lower than the proportion of corresponding age in total AE, while the percentages of patients over 65 years old reporting urinary tract infection, dizziness and condition aggravated were higher than the proportion of corresponding age in total AE, suggesting that older patients may be more prone to these AEs. The absence of reported dizziness and condition aggravated records from Europe and Canada does not mean that AEs did not occur in these regions, most likely due to incomplete or unreported regional records. In addition, we identified 23 AE signals with death outcome, which were relatively low in proportion (less than 0.1%) but still worthy of clinical attention.

In summary, this study investigated and analyzed the AE records of dalfampridine in the FAERS database, and found that the AEs of dalfampridine involved multiple system organ such as nervous system, blood system, urinary system, and some of AEs had gender and age differences. At the same time, we also pointed out some new potential AEs not reported in the instruction and literature. However, there are some shortcomings in this study: 1) This study relied on data recorded by FAERS, so we failed to analyze AE records that were not reported or had incomplete information; 2) the FAERS database was established by the United States FDA, and the recorded data were mainly from the United States, so the analysis in this study could not well distinguish the differences of these adverse events among ethnic groups; 3) this study is a descriptive study, only a description and analysis of existing data, which cannot reveal the causal relationship between AE and drug-used. Nevertheless, this study is of positive significance for the early warning of AEs in dalfampridine. Clinicians should take full consideration of health status of patients and possible AE, and take necessary measures to reduce or avoid the occurrence of AEs.

## Data Availability

The original contributions presented in the study are included in the article/Supplementary Material, further inquiries can be directed to the corresponding authors.
