# Supplementary material for: Post-marketing safety surveillance of dalfampridine for multiple sclerosis using FDA adverse event reporting system
Source: Front Pharmacol. 2023 Sep 14;14:1226086. doi: 10.3389/fphar.2023.1226086 (PMC10538962; doi:10.3389/fphar.2023.1226086)
Supplement: Supplementary file 1 [file Table1.pdf]

Supplementary Material

Post-marketing Safety Surveillance of Dalfampridine for Multiple Sclerosis Using FDA Adverse Event Reporting System

Rui Xiong [1, †], Jing Lei [2, †], Sicen Pan [1], Hong Zhang [3], Yongtao Tong [3], Wei Wu [2, *], Yi Huang [4, *], and Xiaodan Lai [1, *]

[1] Department of Pharmacy, The 958th hospital of Chinese PLA, Chongqing, China
[2] Department of Pharmacy, Daping Hospital, Army Medical University, Chongqing, China
[3] Department of Pharmacy, The 956th hospital of Chinese PLA, Nyingchi, Tibet, China
[4] Biomedical Analysis Center, Army Medical University, Chongqing, China

*** Correspondence:**
Wei Wu: cqcx@163.com
Yi Huang: huangyi@tmmu.edu.cn
Xiaodan Lai: laixiaodan0926@sina.com

Table S1. Top 20 PT of number of AE reports associated with dalfampridine

PT	N (%)	ROR (95% CI)	PRR (χ^2)	IC (IC-2SD)
Urinary tract infection	1061 (8.92)	11.16 (10.47, 11.89)	10.36 (8768.60)	3.33 (3.10)
Dizziness	764 (6.43)	2.39 (2.22, 2.57)	2.31 (578.34)	1.20 (0.96)
Condition aggravated	606 (5.10)	3.58 (3.30, 3.89)	3.47 (1066.43)	1.78 (1.53)
Laboratory test abnormal	516 (4.34)	27.20 (24.83, 29.80)	26.19 (11619.80)	4.61 (4.35)
Seizure	493 (4.15)	5.38 (4.91, 5.89)	5.22 (1667.78)	2.37 (2.11)
Insomnia	478 (4.02)	2.85 (2.60, 3.12)	2.78 (548.15)	1.47 (1.21)
Memory impairment	369 (3.10)	4.26 (3.84, 4.73)	4.17 (883.82)	2.05 (1.78)
Hypoaesthesia	355 (2.99)	3.54 (3.18, 3.93)	3.47 (622.11)	1.78 (1.51)
Flushing	327 (2.75)	5.17 (4.63, 5.77)	5.07 (1057.44)	2.32 (2.05)
Back pain	313 (2.63)	2.16 (1.93, 2.41)	2.13 (188.21)	1.09 (0.81)
Paraesthesia	310 (2.61)	3.08 (2.75, 3.45)	3.03 (420.94)	1.59 (1.32)
Urine analysis abnormal	179 (1.51)	124.45 (104.79, 147.80)	122.81 (15850.60)	6.50 (6.19)
Contusion	142 (1.19)	2.45 (2.07, 2.89)	2.43 (119.54)	1.28 (0.97)
Influenza like illness	138 (1.16)	2.35 (1.99, 2.78)	2.34 (105.38)	1.22 (0.91)
Lymphocyte count decreased	117 (0.98)	9.72 (8.08, 11.69)	9.64 (882.01)	3.23 (2.92)
Head injury	112 (0.94)	5.49 (4.55, 6.62)	5.45 (401.56)	2.43 (2.11)
Central nervous system lesion	110 (0.93)	11.33 (9.37, 13.72)	11.25 (994.74)	3.45 (3.13)
Urinary incontinence	110 (0.93)	6.07 (5.02, 7.33)	6.02 (453.45)	2.57 (2.25)
Cystitis	106 (0.89)	5.06 (4.17, 6.13)	5.03 (337.39)	2.31 (1.99)
Pollakiuria	104 (0.87)	4.24 (3.49, 5.15)	4.22 (252.58)	2.06 (1.74)

Table S2. Detailed information of all positive AE signals associated with dalfampridine

PT	SOC	*a*	*c*	*b*	*d*	N (%)	ROR (95% CI)	PRR (χ^2)	IC (IC-2SD)
Spinal cord injury cauda equina	Injury, poisoning and procedural complications	6	2	13437	4528435	6 (0.05)	1011.04 (204.04, 5009.82)	1010.59 (1512.88)	7.99 (6.99)
CD8 lymphocyte percentage decreased	Investigations	3	1	13440	4528436	3 (0.03)	1010.81 (105.13, 9718.49)	1010.59 (756.44)	7.99 (6.31)
Haemoglobin urine present	Investigations	17	17	13426	4528420	17 (0.14)	337.29 (172.16, 660.78)	336.86 (2846.37)	7.40 (6.84)
CD8 lymphocytes increased	Investigations	6	6	13437	4528431	6 (0.05)	337.01 (108.68, 1045.08)	336.86 (1004.60)	7.40 (6.56)
Somatosensory evoked potentials abnormal	Investigations	6	7	13437	4528430	6 (0.05)	288.87 (97.07, 859.66)	288.74 (926.40)	7.28 (6.46)
CD4 lymphocyte percentage decreased	Investigations	6	8	13437	4528429	6 (0.05)	252.76 (87.69, 728.58)	252.65 (859.38)	7.18 (6.38)
Urine leukocyte esterase positive	Investigations	51	74	13392	4528363	51 (0.43)	233.04 (163.06, 333.06)	232.16 (6949.36)	7.11 (6.70)
B-lymphocyte count abnormal	Investigations	11	16	13432	4528421	11 (0.09)	231.78 (107.55, 499.53)	231.59 (1496.64)	7.10 (6.49)
B-lymphocyte count decreased	Investigations	61	107	13382	4528330	61 (0.51)	192.91 (140.80, 264.32)	192.04 (7383.87)	6.94 (6.55)
T-lymphocyte count increased	Investigations	17	30	13426	4528407	17 (0.14)	191.13 (105.39, 346.61)	190.89 (2049.72)	6.93 (6.41)
CD4 lymphocytes increased	Investigations	15	29	13428	4528408	15 (0.13)	174.43 (93.50, 325.42)	174.24 (1702.89)	6.85 (6.31)
Vitamin B12 abnormal	Investigations	8	17	13435	4528420	8 (0.07)	158.62 (68.44, 367.60)	158.52 (851.53)	6.76 (6.10)
CD8 lymphocytes decreased	Investigations	14	31	13429	4528406	14 (0.12)	152.29 (81.00, 286.33)	152.13 (1448.01)	6.72 (6.18)
Lymphocyte percentage abnormal	Investigations	3	7	13440	4528430	3 (0.03)	144.40 (37.34, 558.49)	144.37 (298.99)	6.66 (5.62)
Urinary sediment abnormal	Investigations	5	13	13438	4528424	5 (0.04)	129.61 (46.20, 363.61)	129.56 (460.67)	6.55 (5.79)
Urine analysis abnormal	Investigations	179	491	13264	4527946	179 (1.51)	124.45 (104.79, 147.80)	122.81 (15850.60)	6.50 (6.19)
Cystitis klebsiella	Infections and infestations	8	24	13435	4528413	8 (0.07)	112.35 (50.47, 250.13)	112.29 (661.78)	6.40 (5.78)
Specific gravity urine abnormal	Investigations	3	9	13440	4528428	3 (0.03)	112.31 (30.40, 414.91)	112.29 (248.17)	6.40 (5.42)
Culture urine positive	Investigations	58	208	13385	4528229	58 (0.49)	94.34 (70.48, 126.27)	93.93 (4170.19)	6.20 (5.83)
T-lymphocyte count decreased	Investigations	27	98	13416	4528339	27 (0.23)	92.99 (60.71, 142.43)	92.81 (1922.52)	6.19 (5.75)
Blood folate increased	Investigations	6	22	13437	4528415	6 (0.05)	91.91 (37.26, 226.72)	91.87 (423.73)	6.18 (5.51)
Urine cannabinoids increased	Investigations	3	11	13440	4528426	3 (0.03)	91.89 (25.63, 329.42)	91.87 (211.87)	6.18 (5.25)
Vitamin B12 increased	Investigations	22	88	13421	4528349	22 (0.19)	84.35 (52.85, 134.63)	84.22 (1447.24)	6.08 (5.62)
Anti-interferon antibody positive	Investigations	3	12	13440	4528425	3 (0.03)	84.23 (23.77, 298.54)	84.22 (197.35)	6.08 (5.17)
Red blood cells urine positive	Investigations	29	123	13414	4528314	29 (0.24)	79.59 (53.09, 119.33)	79.42 (1817.24)	6.01 (5.58)
Nitrite urine present	Investigations	15	68	13428	4528369	15 (0.13)	74.39 (42.52, 130.15)	74.31 (888.78)	5.93 (5.44)
Vitamin B6 increased	Investigations	3	14	13440	4528423	3 (0.03)	72.20 (20.75, 251.27)	72.18 (173.43)	5.90 (5.02)
Basophil percentage increased	Investigations	3	15	13440	4528422	3 (0.03)	67.39 (19.51, 232.80)	67.37 (163.47)	5.82 (4.96)
B-lymphocyte count increased	Investigations	5	26	13438	4528411	5 (0.04)	64.80 (24.88, 168.79)	64.78 (263.34)	5.77 (5.09)
Monocyte count abnormal	Investigations	3	16	13440	4528421	3 (0.03)	63.18 (18.41, 216.84)	63.16 (154.55)	5.74 (4.89)
Urobilinogen urine increased	Investigations	6	34	13437	4528403	6 (0.05)	59.47 (24.96, 141.68)	59.45 (293.06)	5.66 (5.04)
Blood immunoglobulin M decreased	Investigations	17	97	13426	4528340	17 (0.14)	59.11 (35.30, 98.99)	59.04 (825.31)	5.65 (5.18)
White blood cells urine positive	Investigations	41	234	13402	4528203	41 (0.34)	59.20 (42.46, 82.53)	59.02 (1990.08)	5.65 (5.26)
Vitamin B6 deficiency	Metabolism and nutrition disorders	6	36	13437	4528401	6 (0.05)	56.17 (23.66, 133.33)	56.14 (278.55)	5.59 (4.97)
Bacterial test	Investigations	9	62	13434	4528375	9 (0.08)	48.93 (24.31, 98.47)	48.90 (368.76)	5.42 (4.88)
CD4/CD8 ratio decreased	Investigations	3	22	13440	4528415	3 (0.03)	45.95 (13.75, 153.53)	45.94 (116.05)	5.34 (4.55)
Lymphocyte percentage decreased	Investigations	28	214	13415	4528223	28 (0.24)	44.17 (29.78, 65.51)	44.08 (1042.42)	5.29 (4.87)
Lipomatosis	Metabolism and nutrition disorders	6	46	13437	4528391	6 (0.05)	43.96 (18.77, 102.94)	43.94 (222.72)	5.28 (4.68)
Natural killer cell count increased	Investigations	3	23	13440	4528414	3 (0.03)	43.95 (13.19, 146.39)	43.94 (111.36)	5.28 (4.50)
Urinary sediment present	Investigations	11	85	13432	4528352	11 (0.09)	43.63 (23.28, 81.78)	43.59 (405.34)	5.27 (4.76)
Streptococcal urinary tract infection	Infections and infestations	4	31	13439	4528406	4 (0.03)	43.48 (15.35, 123.19)	43.47 (146.99)	5.27 (4.58)
Escherichia urinary tract infection	Infections and infestations	100	844	13343	4527593	100 (0.84)	40.20 (32.65, 49.50)	39.91 (3392.59)	5.16 (4.83)
Transferrin saturation decreased	Investigations	7	63	13436	4528374	7 (0.06)	37.45 (17.15, 81.78)	37.43 (223.38)	5.08 (4.51)
Decreased vibratory sense	Nervous system disorders	10	91	13433	4528346	10 (0.08)	37.04 (19.28, 71.19)	37.02 (315.76)	5.06 (4.55)

SARS-CoV-2 antibody test negative	Investigations	3	28	13440	4528409	3 (0.03)	36.10 (10.97, 118.76)	36.09 (92.46)	5.03 (4.27)
Urinary tract infection pseudomonal	Infections and infestations	13	126	13430	4528311	13 (0.11)	34.79 (19.65, 61.59)	34.76 (386.35)	4.98 (4.50)
Blood immunoglobulin A increased	Investigations	6	66	13437	4528371	6 (0.05)	30.64 (13.28, 70.68)	30.62 (157.61)	4.82 (4.23)
Urinary tract infection staphylococcal	Infections and infestations	6	66	13437	4528371	6 (0.05)	30.64 (13.28, 70.68)	30.62 (157.61)	4.82 (4.23)
Urinary tract infection enterococcal	Infections and infestations	15	166	13428	4528271	15 (0.13)	30.47 (17.96, 51.70)	30.44 (391.70)	4.81 (4.34)
Vitamin D increased	Investigations	8	93	13435	4528344	8 (0.07)	28.99 (14.08, 59.71)	28.98 (198.98)	4.74 (4.21)
Anion gap decreased	Investigations	3	36	13440	4528401	3 (0.03)	28.08 (8.65, 91.19)	28.07 (72.30)	4.70 (3.97)
JC polyomavirus test positive	Investigations	36	445	13407	4527992	36 (0.30)	27.32 (19.45, 38.39)	27.25 (842.34)	4.66 (4.27)
Peroneal nerve palsy	Nervous system disorders	93	1164	13350	4527273	93 (0.78)	27.09 (21.92, 33.49)	26.91 (2149.39)	4.64 (4.31)
Neutrophil percentage increased	Investigations	14	178	13429	4528259	14 (0.12)	26.52 (15.39, 45.71)	26.49 (318.43)	4.62 (4.15)
Laboratory test abnormal	Investigations	516	6636	12927	4521801	516 (4.34)	27.20 (24.83, 29.80)	26.19 (11619.80)	4.61 (4.35)
Monocyte percentage increased	Investigations	8	106	13435	4528331	8 (0.07)	25.44 (12.40, 52.20)	25.42 (174.54)	4.57 (4.04)
Red blood cells urine	Investigations	5	67	13438	4528370	5 (0.04)	25.15 (10.13, 62.40)	25.14 (107.85)	4.55 (3.95)
Proteus test positive	Investigations	4	54	13439	4528383	4 (0.03)	24.96 (9.04, 68.93)	24.95 (85.63)	4.54 (3.90)
Vitamin D abnormal	Investigations	5	68	13438	4528369	5 (0.04)	24.78 (9.99, 61.46)	24.77 (106.24)	4.53 (3.93)
Citrobacter infection	Infections and infestations	5	72	13438	4528365	5 (0.04)	23.40 (9.45, 57.94)	23.39 (100.22)	4.46 (3.86)
Lymphocyte count abnormal	Investigations	13	190	13430	4528247	13 (0.11)	23.07 (13.15, 40.47)	23.05 (256.65)	4.44 (3.96)
Human papilloma virus test positive	Investigations	7	104	13436	4528333	7 (0.06)	22.68 (10.55, 48.77)	22.67 (135.88)	4.41 (3.87)
Mean cell volume abnormal	Investigations	5	76	13438	4528361	5 (0.04)	22.17 (8.97, 54.81)	22.16 (94.80)	4.38 (3.79)
Blood 25-hydroxycholecalciferol decreased	Investigations	3	47	13440	4528390	3 (0.03)	21.51 (6.69, 69.11)	21.50 (55.13)	4.34 (3.63)
Acute disseminated encephalomyelitis	Nervous system disorders	4	63	13439	4528374	4 (0.03)	21.39 (7.79, 58.79)	21.39 (73.10)	4.33 (3.70)
Escherichia test positive	Investigations	18	293	13425	4528144	18 (0.15)	20.72 (12.87, 33.36)	20.69 (317.87)	4.29 (3.85)
Fear of falling	Psychiatric disorders	8	139	13435	4528298	8 (0.07)	19.40 (9.51, 39.57)	19.39 (131.93)	4.20 (3.68)
Urethritis noninfective	Renal and urinary disorders	3	53	13440	4528384	3 (0.03)	19.07 (5.96, 61.04)	19.07 (48.61)	4.18 (3.48)
Neurogenic bladder	Renal and urinary disorders	19	346	13424	4528091	19 (0.16)	18.52 (11.67, 29.40)	18.50 (298.15)	4.14 (3.70)
Cystitis escherichia	Infections and infestations	3	56	13440	4528381	3 (0.03)	18.05 (5.65, 57.67)	18.05 (45.85)	4.10 (3.41)
Bladder dysfunction	Renal and urinary disorders	12	225	13431	4528212	12 (0.10)	17.98 (10.06, 32.15)	17.97 (182.53)	4.10 (3.62)
Creatinine urine increased	Investigations	3	57	13440	4528380	3 (0.03)	17.73 (5.55, 56.63)	17.73 (44.99)	4.08 (3.39)
Hepatitis B surface antibody positive	Investigations	3	59	13440	4528378	3 (0.03)	17.13 (5.37, 54.66)	17.13 (43.36)	4.03 (3.34)
CD4 lymphocytes decreased	Investigations	20	394	13423	4528043	20 (0.17)	17.12 (10.92, 26.84)	17.10 (288.54)	4.03 (3.60)
Crystal urine present	Investigations	4	80	13439	4528357	4 (0.03)	16.85 (6.17, 46.00)	16.84 (56.77)	4.01 (3.38)
Bacterial test positive	Investigations	21	436	13422	4528001	21 (0.18)	16.25 (10.48, 25.18)	16.23 (286.26)	3.96 (3.53)
Urine abnormality	Renal and urinary disorders	33	700	13410	4527737	33 (0.28)	15.92 (11.22, 22.58)	15.88 (439.49)	3.93 (3.53)
Urinary tract infection bacterial	Infections and infestations	25	533	13418	4527904	25 (0.21)	15.83 (10.59, 23.65)	15.80 (331.10)	3.92 (3.51)
Eosinophil percentage increased	Investigations	4	86	13439	4528351	4 (0.03)	15.67 (5.75, 42.72)	15.67 (52.49)	3.91 (3.29)
Trigeminal neuralgia	Nervous system disorders	40	909	13403	4527528	40 (0.34)	14.86 (10.83, 20.41)	14.82 (494.00)	3.83 (3.45)
Urine ketone body present	Investigations	15	345	13428	4528092	15 (0.13)	14.66 (8.74, 24.59)	14.65 (182.78)	3.82 (3.36)
Glucose urine present	Investigations	9	209	13434	4528228	9 (0.08)	14.52 (7.45, 28.29)	14.51 (108.51)	3.80 (3.30)
Albumin urine present	Investigations	3	71	13440	4528366	3 (0.03)	14.24 (4.48, 45.20)	14.23 (35.42)	3.78 (3.10)
Small cell carcinoma	Neoplasms benign, malignant and unspecified (incl cysts and polyps)	3	73	13440	4528364	3 (0.03)	13.85 (4.36, 43.94)	13.84 (34.34)	3.74 (3.06)
Joint instability	Musculoskeletal and connective tissue disorders	10	245	13433	4528192	10 (0.08)	13.76 (7.31, 25.90)	13.75 (113.59)	3.73 (3.24)
Hypertonic bladder	Renal and urinary disorders	18	450	13425	4527987	18 (0.15)	13.49 (8.42, 21.62)	13.47 (199.90)	3.70 (3.26)
Nasal injury	Injury, poisoning and procedural complications	6	150	13437	4528287	6 (0.05)	13.48 (5.96, 30.49)	13.47 (66.63)	3.70 (3.15)

Blood bilirubin decreased	Investigations	5	125	13438	4528312	5 (0.04)	13.48 (5.51, 32.96)	13.47 (55.52)	3.70 (3.12)
Bacteriuria	Infections and infestations	5	126	13438	4528311	5 (0.04)	13.37 (5.47, 32.69)	13.37 (55.03)	3.69 (3.11)
Aspartate aminotransferase decreased	Investigations	4	101	13439	4528336	4 (0.03)	13.34 (4.91, 36.25)	13.34 (43.93)	3.69 (3.07)
Postictal state	Nervous system disorders	9	233	13434	4528204	9 (0.08)	13.02 (6.69, 25.34)	13.01 (96.09)	3.65 (3.15)
Osteochondrosis	Musculoskeletal and connective tissue disorders	6	158	13437	4528279	6 (0.05)	12.80 (5.66, 28.92)	12.79 (62.84)	3.63 (3.08)
Encephalomalacia	Nervous system disorders	4	107	13439	4528330	4 (0.03)	12.60 (4.64, 34.18)	12.59 (41.15)	3.61 (2.99)
Protein urine present	Investigations	30	837	13413	4527600	30 (0.25)	12.10 (8.40, 17.42)	12.07 (294.21)	3.55 (3.15)
Bone contusion	Injury, poisoning and procedural complications	5	140	13438	4528297	5 (0.04)	12.03 (4.93, 29.37)	12.03 (48.83)	3.54 (2.97)
Lacunar stroke	Nervous system disorders	3	84	13440	4528353	3 (0.03)	12.03 (3.80, 38.07)	12.03 (29.30)	3.54 (2.87)
Ankle deformity	Musculoskeletal and connective tissue disorders	3	86	13440	4528351	3 (0.03)	11.75 (3.72, 37.17)	11.75 (28.51)	3.51 (2.84)
Haemorrhagic erosive gastritis	Gastrointestinal disorders	3	89	13440	4528348	3 (0.03)	11.36 (3.59, 35.89)	11.35 (27.41)	3.46 (2.79)
Central nervous system lesion	Nervous system disorders	110	3294	13333	4525143	110 (0.93)	11.33 (9.37, 13.72)	11.25 (994.74)	3.45 (3.13)
Mean cell haemoglobin decreased	Investigations	12	361	13431	4528076	12 (0.10)	11.21 (6.30, 19.92)	11.20 (107.87)	3.44 (2.97)
Bladder disorder	Renal and urinary disorders	64	1965	13379	4526472	64 (0.54)	11.02 (8.59, 14.14)	10.97 (561.97)	3.41 (3.06)
Cervical spinal stenosis	Musculoskeletal and connective tissue disorders	9	282	13434	4528155	9 (0.08)	10.76 (5.54, 20.89)	10.75 (77.14)	3.39 (2.89)
Open fracture	Injury, poisoning and procedural complications	3	95	13440	4528342	3 (0.03)	10.64 (3.37, 33.58)	10.64 (25.39)	3.37 (2.71)
Tongue biting	Nervous system disorders	15	477	13428	4527960	15 (0.13)	10.60 (6.34, 17.73)	10.59 (126.35)	3.36 (2.91)
Haematocrit abnormal	Investigations	5	160	13438	4528277	5 (0.04)	10.53 (4.32, 25.65)	10.53 (41.80)	3.36 (2.78)
Enterococcus test positive	Investigations	6	195	13437	4528242	6 (0.05)	10.37 (4.60, 23.37)	10.36 (49.26)	3.33 (2.79)
Urinary tract infection	Infections and infestations	1061	34515	12382	4493922	1061 (8.92)	11.16 (10.47, 11.89)	10.36 (8768.60)	3.33 (3.10)
Urge incontinence	Renal and urinary disorders	5	164	13438	4528273	5 (0.04)	10.27 (4.22, 25.02)	10.27 (40.60)	3.32 (2.75)
Laboratory test interference	Investigations	5	167	13438	4528270	5 (0.04)	10.09 (4.14, 24.56)	10.09 (39.74)	3.30 (2.73)
Occipital neuralgia	Nervous system disorders	3	102	13440	4528335	3 (0.03)	9.91 (3.14, 31.24)	9.91 (23.34)	3.27 (2.61)
Lymphocyte count decreased	Investigations	117	4087	13326	4524350	117 (0.98)	9.72 (8.08, 11.69)	9.64 (882.01)	3.23 (2.92)
Klebsiella test positive	Investigations	5	175	13438	4528262	5 (0.04)	9.63 (3.96, 23.42)	9.62 (37.57)	3.23 (2.66)
Proteus infection	Infections and infestations	4	140	13439	4528297	4 (0.03)	9.63 (3.56, 26.01)	9.62 (30.06)	3.23 (2.63)
Blood urea nitrogen/creatinine ratio increased	Investigations	3	105	13440	4528332	3 (0.03)	9.63 (3.05, 30.34)	9.62 (22.54)	3.23 (2.57)
Mean cell haemoglobin concentration decreased	Investigations	8	282	13435	4528155	8 (0.07)	9.56 (4.73, 19.31)	9.56 (59.60)	3.22 (2.71)
Invasive lobular breast carcinoma	Neoplasms benign, malignant and unspecified (incl cysts and polyps)	4	142	13439	4528295	4 (0.03)	9.49 (3.51, 25.64)	9.49 (29.55)	3.21 (2.61)
Red cell distribution width increased	Investigations	19	686	13424	4527751	19 (0.16)	9.34 (5.92, 14.74)	9.33 (137.52)	3.19 (2.76)
White matter lesion	Nervous system disorders	6	220	13437	4528217	6 (0.05)	9.19 (4.08, 20.68)	9.19 (42.62)	3.17 (2.62)
Urine odour abnormal	Renal and urinary disorders	33	1221	13410	4527216	33 (0.28)	9.12 (6.45, 12.90)	9.10 (231.87)	3.15 (2.76)
Pseudomonas test positive	Investigations	5	187	13438	4528250	5 (0.04)	9.01 (3.71, 21.90)	9.01 (34.66)	3.14 (2.57)
Blood immunoglobulin G decreased	Investigations	7	264	13436	4528173	7 (0.06)	8.94 (4.22, 18.93)	8.93 (48.04)	3.13 (2.60)
Serum ferritin decreased	Investigations	7	265	13436	4528172	7 (0.06)	8.90 (4.20, 18.86)	8.90 (47.81)	3.12 (2.60)
Chest injury	Injury, poisoning and procedural complications	11	430	13432	4528007	11 (0.09)	8.62 (4.74, 15.69)	8.62 (72.23)	3.08 (2.60)
Distractibility	Psychiatric disorders	6	240	13437	4528197	6 (0.05)	8.42 (3.75, 18.94)	8.42 (38.29)	3.04 (2.50)
Anosognosia	Nervous system disorders	8	325	13435	4528112	8 (0.07)	8.30 (4.11, 16.73)	8.29 (50.07)	3.02 (2.51)
Micturition urgency	Renal and urinary disorders	56	2288	13387	4526149	56 (0.47)	8.28 (6.34, 10.79)	8.24 (348.17)	3.01 (2.66)
Convulsive threshold lowered	Nervous system disorders	3	123	13440	4528314	3 (0.03)	8.22 (2.61, 25.84)	8.22 (18.56)	3.01 (2.35)

Clumsiness	Nervous system disorders	14	577	13429	4527860	14 (0.12)	8.18 (4.81, 13.90)	8.17 (86.06)	3.00 (2.54)
Magnetic resonance imaging abnormal	Investigations	24	992	13419	4527445	24 (0.20)	8.16 (5.44, 12.24)	8.15 (147.02)	3.00 (2.58)
Light chain analysis increased	Investigations	4	166	13439	4528271	4 (0.03)	8.12 (3.01, 21.89)	8.12 (24.38)	2.99 (2.39)
Mean cell volume decreased	Investigations	8	336	13435	4528101	8 (0.07)	8.02 (3.98, 16.18)	8.02 (48.02)	2.97 (2.47)
Adjustment disorder with depressed mood	Psychiatric disorders	3	127	13440	4528310	3 (0.03)	7.96 (2.53, 25.01)	7.96 (17.83)	2.96 (2.31)
Wound infection staphylococcal	Infections and infestations	7	301	13436	4528136	7 (0.06)	7.84 (3.70, 16.58)	7.83 (40.79)	2.94 (2.42)
Retinal vein thrombosis	Eye disorders	3	129	13440	4528308	3 (0.03)	7.84 (2.49, 24.62)	7.83 (17.48)	2.94 (2.29)
Fibula fracture	Injury, poisoning and procedural complications	15	647	13428	4527790	15 (0.13)	7.82 (4.68, 13.05)	7.81 (87.06)	2.94 (2.49)
Adjustment disorder	Psychiatric disorders	6	259	13437	4528178	6 (0.05)	7.81 (3.47, 17.54)	7.80 (34.79)	2.94 (2.39)
Tonic convulsion	Nervous system disorders	5	217	13438	4528220	5 (0.04)	7.76 (3.20, 18.85)	7.76 (28.79)	2.93 (2.36)
Focal dyscognitive seizures	Nervous system disorders	9	394	13434	4528043	9 (0.08)	7.70 (3.98, 14.91)	7.69 (51.26)	2.92 (2.42)
Agraphia	Nervous system disorders	3	136	13440	4528301	3 (0.03)	7.43 (2.37, 23.34)	7.43 (16.34)	2.87 (2.21)
Investigation abnormal	Investigations	4	182	13439	4528255	4 (0.03)	7.41 (2.75, 19.95)	7.40 (21.68)	2.86 (2.26)
Ankle fracture	Injury, poisoning and procedural complications	75	3490	13368	4524947	75 (0.63)	7.27 (5.78, 9.15)	7.24 (395.12)	2.83 (2.49)
Temperature intolerance	General disorders and administration site conditions	49	2327	13394	4526110	49 (0.41)	7.12 (5.36, 9.45)	7.09 (251.32)	2.80 (2.44)
Tibia fracture	Injury, poisoning and procedural complications	22	1050	13421	4527387	22 (0.19)	7.07 (4.63, 10.78)	7.06 (112.07)	2.79 (2.38)
Urinary tract disorder	Renal and urinary disorders	18	870	13425	4527567	18 (0.15)	6.98 (4.37, 11.13)	6.97 (90.19)	2.78 (2.34)
Expanded disability status scale score increased	Investigations	8	392	13435	4528045	8 (0.07)	6.88 (3.41, 13.85)	6.87 (39.36)	2.76 (2.25)
Reflexes abnormal	Nervous system disorders	3	151	13440	4528286	3 (0.03)	6.69 (2.13, 20.99)	6.69 (14.24)	2.72 (2.07)
Blood chloride decreased	Investigations	8	403	13435	4528034	8 (0.07)	6.69 (3.32, 13.47)	6.69 (37.94)	2.72 (2.21)
Band sensation	Nervous system disorders	6	312	13437	4528125	6 (0.05)	6.48 (2.89, 14.54)	6.48 (27.27)	2.67 (2.13)
Thyroxine free increased	Investigations	3	158	13440	4528279	3 (0.03)	6.40 (2.04, 20.05)	6.40 (13.40)	2.65 (2.01)
White blood cell disorder	Blood and lymphatic system disorders	10	541	13433	4527896	10 (0.08)	6.23 (3.33, 11.65)	6.23 (43.08)	2.62 (2.13)
Urethral haemorrhage	Renal and urinary disorders	3	163	13440	4528274	3 (0.03)	6.20 (1.98, 19.43)	6.20 (12.85)	2.61 (1.96)
Erosive duodenitis	Gastrointestinal disorders	4	219	13439	4528218	4 (0.03)	6.15 (2.29, 16.55)	6.15 (16.95)	2.60 (2.01)
Herpes simplex encephalitis	Infections and infestations	3	166	13440	4528271	3 (0.03)	6.09 (1.94, 19.08)	6.09 (12.53)	2.58 (1.94)
Blood thyroid stimulating hormone decreased	Investigations	18	1002	13425	4527435	18 (0.15)	6.06 (3.80, 9.66)	6.05 (74.58)	2.58 (2.14)
Mean cell haemoglobin increased	Investigations	5	279	13438	4528158	5 (0.04)	6.04 (2.49, 14.62)	6.04 (20.64)	2.57 (2.01)
Decreased interest	Psychiatric disorders	22	1230	13421	4527207	22 (0.19)	6.03 (3.96, 9.20)	6.03 (90.61)	2.57 (2.15)
Urinary incontinence	Renal and urinary disorders	110	6151	13333	4522286	110 (0.93)	6.07 (5.02, 7.33)	6.02 (453.45)	2.57 (2.25)
PCO2 decreased	Investigations	3	168	13440	4528269	3 (0.03)	6.02 (1.92, 18.85)	6.02 (12.33)	2.57 (1.92)
Coccydynia	Musculoskeletal and connective tissue disorders	5	282	13438	4528155	5 (0.04)	5.97 (2.47, 14.47)	5.97 (20.34)	2.56 (2.00)
Mean cell volume increased	Investigations	8	454	13435	4527983	8 (0.07)	5.94 (2.95, 11.95)	5.94 (32.27)	2.55 (2.04)
Bladder spasm	Renal and urinary disorders	6	341	13437	4528096	6 (0.05)	5.93 (2.64, 13.29)	5.93 (24.15)	2.55 (2.01)
Red blood cell count abnormal	Investigations	4	229	13439	4528208	4 (0.03)	5.89 (2.19, 15.82)	5.88 (15.94)	2.54 (1.94)
Claustrophobia	Psychiatric disorders	4	231	13439	4528206	4 (0.03)	5.83 (2.17, 15.68)	5.83 (15.75)	2.52 (1.93)
Blood thyroid stimulating hormone abnormal	Investigations	5	290	13438	4528147	5 (0.04)	5.81 (2.40, 14.07)	5.81 (19.56)	2.52 (1.96)
Vulvovaginal candidiasis	Infections and infestations	5	291	13438	4528146	5 (0.04)	5.79 (2.39, 14.02)	5.79 (19.47)	2.51 (1.95)
Status epilepticus	Nervous system disorders	44	2645	13399	4525792	44 (0.37)	5.62 (4.17, 7.57)	5.60 (163.79)	2.47 (2.10)
Occult blood positive	Investigations	9	543	13434	4527894	9 (0.08)	5.59 (2.89, 10.80)	5.58 (33.31)	2.46 (1.97)
Lymphopenia	Blood and lymphatic system disorders	47	2864	13396	4525573	47 (0.40)	5.54 (4.15, 7.40)	5.53 (171.62)	2.45 (2.08)

Glioblastoma multiforme	Neoplasms benign, malignant and unspecified (incl cysts and polyps)	3	185	13440	4528252	3 (0.03)	5.46 (1.75, 17.10)	5.46 (10.76)	2.43 (1.79)
Head injury	Injury, poisoning and procedural complications	112	6917	13331	4521520	112 (0.94)	5.49 (4.55, 6.62)	5.45 (401.56)	2.43 (2.11)
Incontinence	Renal and urinary disorders	35	2178	13408	4526259	35 (0.29)	5.42 (3.88, 7.58)	5.41 (124.00)	2.42 (2.03)
Cerebral atrophy	Nervous system disorders	12	763	13431	4527674	12 (0.10)	5.30 (3.00, 9.38)	5.30 (41.20)	2.39 (1.92)
Magnetic resonance imaging head abnormal	Investigations	5	318	13438	4528119	5 (0.04)	5.30 (2.19, 12.82)	5.30 (17.16)	2.39 (1.83)
Protein urine	Investigations	3	191	13440	4528246	3 (0.03)	5.29 (1.69, 16.56)	5.29 (10.28)	2.39 (1.74)
Neutrophil count abnormal	Investigations	7	446	13436	4527991	7 (0.06)	5.29 (2.51, 11.16)	5.29 (23.96)	2.38 (1.87)
General symptom	General disorders and administration site conditions	23	1472	13420	4526965	23 (0.19)	5.27 (3.49, 7.96)	5.26 (78.23)	2.38 (1.96)
Vitamin D decreased	Investigations	25	1605	13418	4526832	25 (0.21)	5.25 (3.54, 7.80)	5.25 (84.65)	2.37 (1.97)
Lip injury	Injury, poisoning and procedural complications	4	258	13439	4528179	4 (0.03)	5.22 (1.95, 14.03)	5.22 (13.45)	2.37 (1.78)
Seizure	Nervous system disorders	493	31819	12950	4496618	493 (4.15)	5.38 (4.91, 5.89)	5.22 (1667.78)	2.37 (2.11)
Neutrophil count increased	Investigations	37	2405	13406	4526032	37 (0.31)	5.19 (3.75, 7.19)	5.18 (123.07)	2.36 (1.97)
Energy increased	General disorders and administration site conditions	17	1110	13426	4527327	17 (0.14)	5.16 (3.20, 8.34)	5.16 (56.15)	2.35 (1.91)
Eye contusion	Injury, poisoning and procedural complications	7	460	13436	4527997	7 (0.06)	5.13 (2.43, 10.82)	5.13 (22.90)	2.34 (1.82)
Cerebral thrombosis	Nervous system disorders	10	659	13433	4527778	10 (0.08)	5.11 (2.74, 9.55)	5.11 (32.58)	2.34 (1.85)
Flushing	Vascular disorders	327	21730	13116	4506707	327 (2.75)	5.17 (4.63, 5.77)	5.07 (1057.44)	2.32 (2.05)
Lymphocyte count increased	Investigations	10	670	13433	4527767	10 (0.08)	5.03 (2.69, 9.40)	5.03 (31.80)	2.31 (1.83)
Cystitis	Infections and infestations	106	7104	13337	4521333	106 (0.89)	5.06 (4.17, 6.13)	5.03 (337.39)	2.31 (1.99)
Febrile infection	Infections and infestations	3	202	13440	4528235	3 (0.03)	5.00 (1.60, 15.65)	5.00 (9.47)	2.31 (1.66)
Lymphoedema	Vascular disorders	19	1310	13424	4527127	19 (0.16)	4.89 (3.11, 7.70)	4.89 (57.90)	2.27 (1.84)
Lower limb fracture	Injury, poisoning and procedural complications	58	4001	13385	4524436	58 (0.49)	4.90 (3.78, 6.35)	4.88 (176.71)	2.27 (1.92)
Vitamin D deficiency	Metabolism and nutrition disorders	26	1808	13417	4526629	26 (0.22)	4.85 (3.29, 7.15)	4.84 (78.22)	2.26 (1.85)
Seborrhoeic keratosis	Neoplasms benign, malignant and unspecified (incl cysts and polyps)	4	281	13439	4528156	4 (0.03)	4.80 (1.79, 12.87)	4.80 (11.85)	2.25 (1.66)
Blood chloride increased	Investigations	4	281	13439	4528156	4 (0.03)	4.80 (1.79, 12.87)	4.80 (11.85)	2.25 (1.66)
Meningitis cryptococcal	Infections and infestations	6	423	13437	4528014	6 (0.05)	4.78 (2.13, 10.70)	4.78 (17.68)	2.24 (1.70)
Streptococcus test positive	Investigations	4	284	13439	4528153	4 (0.03)	4.75 (1.77, 12.73)	4.74 (11.66)	2.23 (1.64)
Infusion site bruising	General disorders and administration site conditions	3	213	13440	4528224	3 (0.03)	4.75 (1.52, 14.83)	4.74 (8.74)	2.23 (1.59)
Blood sodium increased	Investigations	7	499	13436	4527938	7 (0.06)	4.73 (2.24, 9.97)	4.73 (20.28)	2.22 (1.71)
Concussion	Injury, poisoning and procedural complications	21	1504	13422	4526933	21 (0.18)	4.71 (3.06, 7.25)	4.70 (60.42)	2.22 (1.80)
Vertigo positional	Ear and labyrinth disorders	5	360	13438	4528077	5 (0.04)	4.68 (1.94, 11.31)	4.68 (14.26)	2.21 (1.65)
Progressive multifocal leukoencephalopathy	Infections and infestations	31	2235	13412	4526202	31 (0.26)	4.68 (3.28, 6.67)	4.67 (88.30)	2.21 (1.82)
Pelvic fracture	Injury, poisoning and procedural complications	3	217	13440	4528220	3 (0.03)	4.66 (1.49, 14.56)	4.66 (8.50)	2.20 (1.56)
Joint lock	Musculoskeletal and connective tissue disorders	8	584	13435	4527853	8 (0.07)	4.62 (2.30, 9.28)	4.61 (22.35)	2.19 (1.69)
Skin abrasion	Injury, poisoning and procedural complications	19	1389	13424	4527048	19 (0.16)	4.61 (2.93, 7.26)	4.61 (52.97)	2.19 (1.76)
Costochondritis	Musculoskeletal and connective tissue disorders	4	293	13439	4528144	4 (0.03)	4.60 (1.71, 12.34)	4.60 (11.11)	2.19 (1.60)

Invasive ductal breast carcinoma	Neoplasms benign, malignant and unspecified (incl cysts and polyps)	10	736	13433	4527701	10 (0.08)	4.58 (2.45, 8.55)	4.58 (27.58)	2.18 (1.70)
High density lipoprotein increased	Investigations	3	221	13440	4528216	3 (0.03)	4.57 (1.46, 14.29)	4.57 (8.26)	2.18 (1.54)
Breast cyst	Reproductive system and breast disorders	5	370	13438	4528067	5 (0.04)	4.55 (1.88, 11.01)	4.55 (13.68)	2.17 (1.61)
Ureterolithiasis	Renal and urinary disorders	3	222	13440	4528215	3 (0.03)	4.55 (1.46, 14.23)	4.55 (8.21)	2.17 (1.53)
Blood urea decreased	Investigations	3	223	13440	4528214	3 (0.03)	4.53 (1.45, 14.16)	4.53 (8.15)	2.17 (1.52)
Urosepsis	Infections and infestations	29	2166	13414	4526271	29 (0.24)	4.52 (3.13, 6.52)	4.51 (78.22)	2.16 (1.76)
Humerus fracture	Injury, poisoning and procedural complications	15	1126	13428	4527311	15 (0.13)	4.49 (2.70, 7.48)	4.49 (40.13)	2.15 (1.70)
Protein total decreased	Investigations	8	602	13435	4527835	8 (0.07)	4.48 (2.23, 9.00)	4.48 (21.32)	2.15 (1.64)
Thrombocytopenic purpura	Blood and lymphatic system disorders	4	301	13439	4528136	4 (0.03)	4.48 (1.67, 12.01)	4.48 (10.66)	2.15 (1.56)
Wrist fracture	Injury, poisoning and procedural complications	35	2635	13408	4525802	35 (0.29)	4.48 (3.21, 6.26)	4.47 (93.24)	2.15 (1.76)
Simple partial seizures	Nervous system disorders	3	228	13440	4528209	3 (0.03)	4.43 (1.42, 13.85)	4.43 (7.87)	2.13 (1.49)
Skin laceration	Injury, poisoning and procedural complications	56	4269	13387	4524168	56 (0.47)	4.43 (3.40, 5.77)	4.42 (146.35)	2.13 (1.77)
Cystitis noninfective	Renal and urinary disorders	3	229	13440	4528208	3 (0.03)	4.41 (1.41, 13.79)	4.41 (7.82)	2.13 (1.49)
Micturition disorder	Renal and urinary disorders	9	689	13434	4527748	9 (0.08)	4.40 (2.28, 8.50)	4.40 (23.35)	2.12 (1.63)
Haematocrit decreased	Investigations	49	3810	13394	4524627	49 (0.41)	4.34 (3.28, 5.76)	4.33 (124.11)	2.10 (1.74)
Ligament sprain	Injury, poisoning and procedural complications	24	1871	13419	4526566	24 (0.20)	4.33 (2.89, 6.47)	4.32 (60.51)	2.10 (1.69)
Face injury	Injury, poisoning and procedural complications	10	782	13433	4527655	10 (0.08)	4.31 (2.31, 8.04)	4.31 (25.08)	2.09 (1.61)
Infection susceptibility increased	Infections and infestations	5	392	13438	4528045	5 (0.04)	4.30 (1.78, 10.39)	4.30 (12.49)	2.09 (1.53)
Initial insomnia	Psychiatric disorders	20	1569	13423	4526868	20 (0.17)	4.30 (2.76, 6.68)	4.29 (49.92)	2.09 (1.66)
Functional gastrointestinal disorder	Gastrointestinal disorders	10	790	13433	4527647	10 (0.08)	4.27 (2.29, 7.96)	4.26 (24.68)	2.08 (1.60)
Pollakiuria	Renal and urinary disorders	104	8307	13339	4520130	104 (0.87)	4.24 (3.49, 5.15)	4.22 (252.58)	2.06 (1.74)
Craniocerebral injury	Injury, poisoning and procedural complications	9	724	13434	4527713	9 (0.08)	4.19 (2.17, 8.09)	4.19 (21.57)	2.05 (1.56)
Extensor plantar response	Nervous system disorders	3	242	13440	4528195	3 (0.03)	4.18 (1.34, 13.04)	4.18 (7.16)	2.05 (1.41)
Weight bearing difficulty	Musculoskeletal and connective tissue disorders	8	646	13435	4527791	8 (0.07)	4.17 (2.08, 8.38)	4.17 (19.06)	2.05 (1.54)
Memory impairment	Nervous system disorders	369	29817	13074	4498620	369 (3.10)	4.26 (3.84, 4.73)	4.17 (883.82)	2.05 (1.78)
Blood creatinine decreased	Investigations	6	494	13437	4527943	6 (0.05)	4.09 (1.83, 9.16)	4.09 (13.85)	2.02 (1.49)
Anxiety disorder	Psychiatric disorders	8	659	13435	4527778	8 (0.07)	4.09 (2.04, 8.22)	4.09 (18.45)	2.02 (1.52)
Carotid artery disease	Nervous system disorders	3	248	13440	4528189	3 (0.03)	4.08 (1.31, 12.73)	4.07 (6.88)	2.01 (1.38)
Neck injury	Injury, poisoning and procedural complications	7	594	13436	4527843	7 (0.06)	3.97 (1.88, 8.37)	3.97 (15.37)	1.98 (1.46)
Blood creatinine abnormal	Investigations	8	680	13435	4527757	8 (0.07)	3.96 (1.97, 7.96)	3.96 (17.52)	1.97 (1.47)
Monocyte count increased	Investigations	8	680	13435	4527757	8 (0.07)	3.96 (1.97, 7.96)	3.96 (17.52)	1.97 (1.47)
Throat clearing	Respiratory, thoracic and mediastinal disorders	8	683	13435	4527754	8 (0.07)	3.95 (1.97, 7.93)	3.95 (17.39)	1.97 (1.46)
Intraductal proliferative breast lesion	Neoplasms benign, malignant and unspecified (incl cysts and polyps)	4	343	13439	4528094	4 (0.03)	3.93 (1.47, 10.53)	3.93 (8.63)	1.96 (1.37)
Generalised tonic-clonic seizure	Nervous system disorders	69	5956	13374	4522481	69 (0.58)	3.92 (3.09, 4.97)	3.90 (147.45)	1.95 (1.61)
Spinal cord disorder	Nervous system disorders	4	348	13439	4528089	4 (0.03)	3.87 (1.45, 10.38)	3.87 (8.42)	1.94 (1.35)

Vitamin B12 deficiency	Metabolism and nutrition disorders	9	786	13434	4527651	9 (0.08)	3.86 (2.00, 7.45)	3.86 (18.84)	1.94 (1.44)
Dysgraphia	Nervous system disorders	16	1405	13427	4527032	16 (0.13)	3.84 (2.34, 6.29)	3.84 (33.18)	1.93 (1.49)
Electric shock sensation	Nervous system disorders	4	354	13439	4528083	4 (0.03)	3.81 (1.42, 10.20)	3.81 (8.18)	1.92 (1.33)
Urinary hesitation	Renal and urinary disorders	6	532	13437	4527905	6 (0.05)	3.80 (1.70, 8.50)	3.80 (12.24)	1.91 (1.38)
Epilepsy	Nervous system disorders	69	6195	13374	4522242	69 (0.58)	3.77 (2.97, 4.78)	3.75 (137.93)	1.90 (1.55)
Decubitus ulcer	Skin and subcutaneous tissue disorders	18	1620	13425	4526817	18 (0.15)	3.75 (2.35, 5.96)	3.74 (35.80)	1.89 (1.46)
Hip fracture	Injury, poisoning and procedural complications	78	7207	13365	4521230	78 (0.66)	3.66 (2.93, 4.58)	3.65 (148.40)	1.85 (1.52)
Alanine aminotransferase abnormal	Investigations	4	371	13439	4528066	4 (0.03)	3.63 (1.36, 9.73)	3.63 (7.55)	1.85 (1.26)
Facial pain	General disorders and administration site conditions	20	1891	13423	4526546	20 (0.17)	3.57 (2.29, 5.54)	3.56 (36.50)	1.82 (1.40)
Femoral neck fracture	Injury, poisoning and procedural complications	14	1336	13429	4527101	14 (0.12)	3.53 (2.09, 5.98)	3.53 (25.13)	1.81 (1.36)
Full blood count abnormal	Investigations	24	2296	13419	4526141	24 (0.20)	3.53 (2.36, 5.27)	3.52 (42.90)	1.81 (1.40)
Dysuria	Renal and urinary disorders	84	8038	13359	4520399	84 (0.71)	3.54 (2.85, 4.39)	3.52 (150.27)	1.80 (1.47)
Intervertebral disc disorder	Musculoskeletal and connective tissue disorders	9	866	13434	4527571	9 (0.08)	3.50 (1.82, 6.76)	3.50 (15.92)	1.80 (1.31)
Foot fracture	Injury, poisoning and procedural complications	43	4155	13400	4524282	43 (0.36)	3.49 (2.59, 4.72)	3.49 (75.53)	1.79 (1.42)
Hypoaesthesia	Nervous system disorders	355	34477	13088	4493960	355 (2.99)	3.54 (3.18, 3.93)	3.47 (622.11)	1.78 (1.51)
Condition aggravated	General disorders and administration site conditions	606	58896	12837	4469541	606 (5.10)	3.58 (3.30, 3.89)	3.47 (1066.43)	1.78 (1.53)
Glycosylated haemoglobin increased	Investigations	43	4233	13400	4524204	43 (0.36)	3.43 (2.54, 4.63)	3.42 (73.04)	1.76 (1.39)
Facial bones fracture	Injury, poisoning and procedural complications	10	986	13433	4527451	10 (0.08)	3.42 (1.83, 6.37)	3.42 (16.92)	1.76 (1.28)
Anal incontinence	Gastrointestinal disorders	16	1588	13427	4526849	16 (0.13)	3.40 (2.08, 5.56)	3.39 (26.76)	1.75 (1.31)
Intracranial aneurysm	Nervous system disorders	9	908	13434	4527529	9 (0.08)	3.34 (1.73, 6.44)	3.34 (14.60)	1.73 (1.24)
Lung cancer metastatic	Neoplasms benign, malignant and unspecified (incl cysts and polyps)	7	707	13436	4527730	7 (0.06)	3.34 (1.58, 7.03)	3.34 (11.34)	1.73 (1.21)
Joint injury	Injury, poisoning and procedural complications	42	4269	13401	4524168	42 (0.35)	3.32 (2.45, 4.50)	3.31 (67.27)	1.72 (1.35)
Frustration tolerance decreased	Psychiatric disorders	15	1525	13428	4526912	15 (0.13)	3.32 (1.99, 5.52)	3.31 (24.00)	1.72 (1.27)
Injection site scar	General disorders and administration site conditions	5	512	13438	4527925	5 (0.04)	3.29 (1.36, 7.94)	3.29 (7.89)	1.71 (1.15)
Creatinine renal clearance decreased	Investigations	9	924	13434	4527513	9 (0.08)	3.28 (1.70, 6.33)	3.28 (14.14)	1.70 (1.21)
Graves' disease	Endocrine disorders	6	626	13437	4527811	6 (0.05)	3.23 (1.45, 7.22)	3.23 (9.14)	1.68 (1.15)
Onychomycosis	Infections and infestations	6	641	13437	4527796	6 (0.05)	3.15 (1.41, 7.05)	3.15 (8.74)	1.65 (1.12)
Hyperventilation	Respiratory, thoracic and mediastinal disorders	12	1285	13431	4527152	12 (0.10)	3.15 (1.78, 5.56)	3.15 (17.41)	1.64 (1.18)
Joint dislocation	Injury, poisoning and procedural complications	19	2038	13424	4526399	19 (0.16)	3.14 (2.00, 4.94)	3.14 (27.48)	1.64 (1.21)
Polymenorrhoea	Reproductive system and breast disorders	5	540	13438	4527897	5 (0.04)	3.12 (1.29, 7.53)	3.12 (7.13)	1.63 (1.08)
Sensory loss	Nervous system disorders	17	1847	13426	4526590	17 (0.14)	3.10 (1.92, 5.00)	3.10 (23.98)	1.62 (1.19)
Hand fracture	Injury, poisoning and procedural complications	14	1526	13429	4526911	14 (0.12)	3.09 (1.83, 5.24)	3.09 (19.62)	1.62 (1.17)
Thyroid mass	Endocrine disorders	5	554	13438	4527883	5 (0.04)	3.04 (1.26, 7.34)	3.04 (6.79)	1.60 (1.04)
Bladder pain	Renal and urinary disorders	6	667	13437	4527770	6 (0.05)	3.03 (1.36, 6.77)	3.03 (8.09)	1.59 (1.06)
Paraesthesia	Nervous system disorders	310	34470	13133	4493967	310 (2.61)	3.08 (2.75, 3.45)	3.03 (420.94)	1.59 (1.32)

Wound haemorrhage	Injury, poisoning and procedural complications	7	795	13436	4527642	7 (0.06)	2.97 (1.41, 6.25)	2.97 (9.04)	1.56 (1.05)
Meniscus injury	Injury, poisoning and procedural complications	7	803	13436	4527634	7 (0.06)	2.94 (1.40, 6.18)	2.94 (8.86)	1.55 (1.03)
Tendon injury	Injury, poisoning and procedural complications	5	576	13438	4527861	5 (0.04)	2.92 (1.21, 7.06)	2.92 (6.28)	1.54 (0.99)
Sensory disturbance	Nervous system disorders	28	3234	13415	4525203	28 (0.24)	2.92 (2.01, 4.24)	2.92 (34.99)	1.54 (1.14)
Ill-defined disorder	General disorders and administration site conditions	51	5903	13392	4522534	51 (0.43)	2.92 (2.21, 3.85)	2.91 (63.49)	1.53 (1.17)
Limb injury	Injury, poisoning and procedural complications	45	5244	13398	4523193	45 (0.38)	2.90 (2.16, 3.89)	2.89 (55.24)	1.52 (1.16)
Upper limb fracture	Injury, poisoning and procedural complications	41	4790	13402	4523647	41 (0.34)	2.89 (2.12, 3.93)	2.88 (50.06)	1.52 (1.15)
Atrioventricular block first degree	Cardiac disorders	11	1301	13432	4527136	11 (0.09)	2.85 (1.57, 5.16)	2.85 (13.09)	1.50 (1.03)
Therapeutic response changed	General disorders and administration site conditions	6	710	13437	4527727	6 (0.05)	2.85 (1.27, 6.36)	2.85 (7.13)	1.50 (0.97)
Neuralgia	Nervous system disorders	44	5264	13399	4523173	44 (0.37)	2.82 (2.10, 3.80)	2.82 (51.15)	1.49 (1.12)
Cognitive disorder	Nervous system disorders	88	10533	13355	4517904	88 (0.74)	2.83 (2.29, 3.49)	2.81 (102.32)	1.49 (1.16)
Ligament rupture	Injury, poisoning and procedural complications	8	962	13435	4527475	8 (0.07)	2.80 (1.40, 5.62)	2.80 (9.19)	1.48 (0.98)
Insomnia	Psychiatric disorders	478	57881	12965	4470556	478 (4.02)	2.85 (2.60, 3.12)	2.78 (548.15)	1.47 (1.21)
Scoliosis	Musculoskeletal and connective tissue disorders	7	850	13436	4527587	7 (0.06)	2.78 (1.32, 5.84)	2.77 (7.88)	1.46 (0.95)
Pelvic fracture	Injury, poisoning and procedural complications	14	1707	13429	4526730	14 (0.12)	2.76 (1.63, 4.68)	2.76 (15.62)	1.46 (1.01)
Poor venous access	Vascular disorders	15	1835	13428	4526602	15 (0.13)	2.76 (1.66, 4.58)	2.75 (16.62)	1.45 (1.01)
Blood urine present	Investigations	33	4044	13410	4524393	33 (0.28)	2.75 (1.95, 3.88)	2.75 (36.45)	1.45 (1.06)
Spinal osteoarthritis	Musculoskeletal and connective tissue disorders	12	1474	13431	4526963	12 (0.10)	2.74 (1.55, 4.84)	2.74 (13.18)	1.45 (0.98)
Rib fracture	Injury, poisoning and procedural complications	35	4432	13408	4524005	35 (0.29)	2.66 (1.91, 3.72)	2.66 (36.02)	1.40 (1.02)
Nocturia	Renal and urinary disorders	16	2073	13427	4526364	16 (0.13)	2.60 (1.59, 4.26)	2.60 (15.64)	1.37 (0.93)
Intervertebral disc protrusion	Musculoskeletal and connective tissue disorders	32	4167	13411	4524270	32 (0.27)	2.59 (1.83, 3.67)	2.59 (30.94)	1.36 (0.97)
Platelet count abnormal	Investigations	8	1043	13435	4527394	8 (0.07)	2.58 (1.29, 5.18)	2.58 (7.71)	1.36 (0.86)
Low density lipoprotein increased	Investigations	12	1573	13431	4526864	12 (0.10)	2.57 (1.46, 4.54)	2.57 (11.42)	1.35 (0.89)
Klebsiella infection	Infections and infestations	8	1051	13435	4527386	8 (0.07)	2.57 (1.28, 5.14)	2.56 (7.58)	1.35 (0.85)
Basal cell carcinoma	Neoplasms benign, malignant and unspecified (incl cysts and polyps)	23	3045	13420	4525392	23 (0.19)	2.55 (1.69, 3.84)	2.54 (21.41)	1.34 (0.93)
Kidney infection	Infections and infestations	31	4105	13412	4524332	31 (0.26)	2.55 (1.79, 3.63)	2.54 (28.86)	1.34 (0.95)
Fungal skin infection	Infections and infestations	6	797	13437	4527640	6 (0.05)	2.54 (1.14, 5.66)	2.54 (5.54)	1.34 (0.80)
Red blood cell count decreased	Investigations	41	5450	13402	4522987	41 (0.34)	2.54 (1.87, 3.45)	2.53 (37.84)	1.33 (0.96)
Spinal stenosis	Musculoskeletal and connective tissue disorders	8	1068	13435	4527369	8 (0.07)	2.52 (1.26, 5.06)	2.52 (7.30)	1.33 (0.83)
Dysarthria	Nervous system disorders	72	9714	13371	4518723	72 (0.61)	2.50 (1.99, 3.16)	2.50 (64.27)	1.31 (0.97)
Sciatica	Nervous system disorders	19	2577	13424	4525860	19 (0.16)	2.49 (1.58, 3.90)	2.48 (16.73)	1.31 (0.88)
Feeling jittery	General disorders and administration	29	3934	13414	4524503	29 (0.24)	2.49 (1.72, 3.58)	2.48 (25.53)	1.31 (0.91)

	site conditions								
Nephrolithiasis	Renal and urinary disorders	63	8583	13380	4519854	63 (0.53)	2.48 (1.93, 3.18)	2.47 (54.95)	1.30 (0.95)
Contusion	Injury, poisoning and procedural complications	142	19659	13301	4508778	142 (1.19)	2.45 (2.07, 2.89)	2.43 (119.54)	1.28 (0.97)
Feeling of despair	Psychiatric disorders	7	970	13436	4527467	7 (0.06)	2.43 (1.16, 5.12)	2.43 (5.86)	1.28 (0.76)
Discomfort	General disorders and administration site conditions	9	1256	13434	4527181	9 (0.08)	2.41 (1.25, 4.65)	2.41 (7.40)	1.27 (0.78)
Rotator cuff syndrome	Musculoskeletal and connective tissue disorders	18	2513	13425	4525924	18 (0.15)	2.41 (1.52, 3.84)	2.41 (14.79)	1.26 (0.83)
Dysphemia	Psychiatric disorders	7	988	13436	4527449	7 (0.06)	2.39 (1.13, 5.02)	2.39 (5.60)	1.25 (0.73)
Chromaturia	Renal and urinary disorders	35	4969	13408	4523468	35 (0.29)	2.38 (1.70, 3.31)	2.37 (27.63)	1.24 (0.86)
Depressed mood	Psychiatric disorders	87	12366	13356	4516071	87 (0.73)	2.38 (1.93, 2.94)	2.37 (68.60)	1.24 (0.91)
Coronavirus infection	Infections and infestations	9	1284	13434	4527153	9 (0.08)	2.36 (1.23, 4.55)	2.36 (7.02)	1.23 (0.74)
Aphasia	Nervous system disorders	51	7345	13392	4521092	51 (0.43)	2.34 (1.78, 3.09)	2.34 (38.89)	1.22 (0.86)
Influenza like illness	General disorders and administration site conditions	138	19885	13305	4508552	138 (1.16)	2.35 (1.99, 2.78)	2.34 (105.38)	1.22 (0.91)
Vaginal infection	Infections and infestations	7	1010	13436	4527427	7 (0.06)	2.34 (1.11, 4.91)	2.33 (5.31)	1.22 (0.70)
Dizziness	Nervous system disorders	764	111385	12679	4417052	764 (6.43)	2.39 (2.22, 2.57)	2.31 (578.34)	1.20 (0.96)
Urinary retention	Renal and urinary disorders	51	7484	13392	4520953	51 (0.43)	2.30 (1.75, 3.03)	2.30 (37.10)	1.19 (0.83)
Back injury	Injury, poisoning and procedural complications	13	1937	13430	4526500	13 (0.11)	2.26 (1.31, 3.90)	2.26 (9.08)	1.17 (0.71)
Feeling drunk	General disorders and administration site conditions	11	1665	13432	4526772	11 (0.09)	2.23 (1.23, 4.03)	2.23 (7.38)	1.15 (0.68)
Bursitis	Musculoskeletal and connective tissue disorders	12	1836	13431	4526601	12 (0.10)	2.20 (1.25, 3.89)	2.20 (7.82)	1.13 (0.67)
Speech disorder	Nervous system disorders	78	12128	13365	4516309	78 (0.66)	2.17 (1.74, 2.72)	2.17 (48.81)	1.11 (0.77)
Hypotonia	Nervous system disorders	11	1715	13432	4526722	11 (0.09)	2.16 (1.19, 3.91)	2.16 (6.82)	1.11 (0.64)
Peripheral coldness	Vascular disorders	19	2965	13424	4525472	19 (0.16)	2.16 (1.38, 3.39)	2.16 (11.75)	1.11 (0.68)
Immune reconstitution inflammatory syndrome	Immune system disorders	9	1412	13434	4527025	9 (0.08)	2.15 (1.12, 4.14)	2.15 (5.48)	1.10 (0.61)
Psychomotor hyperactivity	Nervous system disorders	20	3163	13423	4525274	20 (0.17)	2.13 (1.37, 3.31)	2.13 (11.92)	1.09 (0.66)
Back pain	Musculoskeletal and connective tissue disorders	313	49532	13130	4478905	313 (2.63)	2.16 (1.93, 2.41)	2.13 (188.21)	1.09 (0.81)
Feeling of body temperature change	General disorders and administration site conditions	9	1435	13434	4527002	9 (0.08)	2.11 (1.10, 4.07)	2.11 (5.24)	1.07 (0.59)
Glomerular filtration rate decreased	Investigations	16	2562	13427	4525875	16 (0.13)	2.11 (1.29, 3.44)	2.10 (9.21)	1.07 (0.63)
Vertigo	Ear and labyrinth disorders	88	14110	13355	4514327	88 (0.74)	2.11 (1.71, 2.60)	2.10 (50.61)	1.07 (0.74)
Middle insomnia	Psychiatric disorders	23	3738	13420	4524699	23 (0.19)	2.07 (1.38, 3.13)	2.07 (12.70)	1.05 (0.63)
Blood iron decreased	Investigations	12	1959	13431	4526478	12 (0.10)	2.06 (1.17, 3.64)	2.06 (6.54)	1.04 (0.58)
Burning sensation	Nervous system disorders	98	16008	13345	4512429	98 (0.82)	2.07 (1.70, 2.53)	2.06 (53.48)	1.04 (0.72)
Femur fracture	Injury, poisoning and procedural complications	43	7128	13400	4521309	43 (0.36)	2.04 (1.51, 2.75)	2.03 (22.44)	1.02 (0.65)
